# Impact of climate change on wood and woodworkers—*Cryptostroma corticale* (sooty bark disease): A risk factor for trees and exposed employees

**DOI:** 10.3389/fpubh.2022.973686

**Published:** 2022-10-18

**Authors:** Sabine Kespohl, Janett Riebesehl, Jörg Grüner, Monika Raulf

**Affiliations:** ^1^Institute for Prevention and Occupational Medicine of the DGUV, Institute of the Ruhr-University Bochum (IPA), Bochum, Germany; ^2^Julius Kühn Institute (JKI)—Federal Research Centre for Cultivated Plants, Institute for Plant Protection in Horticulture and Forests, Braunschweig, Germany; ^3^Department of Forest Protection, Forest Research Institute of Baden-Württemberg (FVA), Freiburg, Germany

**Keywords:** fungal antigens, occupational disease, plant pathogen, respiratory complaints, wood workers, serological IgG

## Abstract

Climate changes have promoted an increased fungal infection of maple trees with *Cryptostroma corticale*, the causative agent of sooty bark disease. The hosts of *C. corticale* are maples, and since the early 2000s the fungus has been appearing more frequently in European forests, due to the droughts and hot summers of recent years. Infestation by *C. corticale* discolors the wood and makes it unusable for further processing, which leads to considerable economic damage in the timber industry. Therefore, the occurrence and spread of sooty bark disease raise serious problems. In addition to forestry and economic problems, the conidiospores of *C. corticale* can also cause health problems in exposed wood workers and they can trigger hypersensitivity pneumonitis (HP). Since the spores, which are deposited over the entire area under the bark of infected trees, can spread during processing, exposed workers must take special precautions to protect themselves against exposure. If an occupational disease is nevertheless suspected following exposure to *C. corticale*, valid diagnostics are required to confirm possible HP and derive appropriate therapies and exposure reduction or avoidance. Diagnosis of HP is based on several criteria, one of them is the detection of specific IgG in patient's serum against the potentially triggering antigens, in this case *C. corticale* antigens. To produce a diagnostic tool to measure *C. corticale* specific IgG, which is not commercially available so far, spores and mycelial material from ITS-sequenced strains of *C. corticale* was prepared and analyzed. These biochemically characterized extracts of spore and mycelial antigens were biotinylated and coupled to Streptavidin-ImmunoCAPs. To validate these diagnostic test tools the first step is to measure the concentration of *C. corticale* specific IgG in sera of healthy non-exposed and healthy exposed subjects to establish cut-off values. Suitable participants were recruited and the individual exposure to *C. corticale* and symptoms experienced during or after working with infected maple trees were recorded using questionnaires. Finally, diagnostic tools for serological testing in suspected cases of HP by *C. corticale* were created and evaluated. The following article provides recommendations for the treatment and disposal of infected damaged wood and for occupational health protection procedures. Secondly, the diagnosis of HP induced by exposure to *C. corticale* as an occupational disease is described including the verification of newly developed serological test tools for antigens of *C. corticale*.

## Introduction

### Cryptostroma corticale

**Figure 1:**
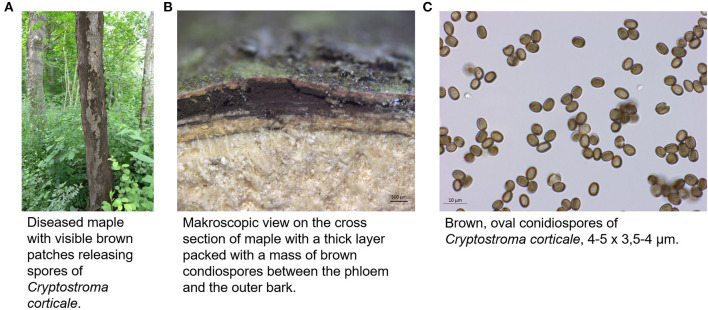
Appearance of *C. corticale* **(A)** on maple tree, **(B)** under the bark of maple trunk, **(C)** light microscopy of conidiospores (JKI-GFF-2019-006).

The causal agent of the sooty bark disease, *Cryptostroma corticale* (Ellis & Everh.) P. H. Greg. & S. Waller, is an ascomycetous fungus out of the family Graphostromataceae (Xylariales) (1). First discovered in 1889 in North America (2), it was probably introduced in 1945 *via* the port of London to Europe (3). *Cryptostroma corticale* can occur as endophyte in its living host substrate. If the host tree is weakened by environmental factors, i.e., water stress, *C. corticale* can change its lifestyle into a parasitic stage which leads in most cases to the death of the tree. Afterwards, the fungus lives saprotrophically on the wood. This is especially the case on *Acer pseudoplatanus* L. in Europe. On other woody substrates, *C. corticale* is mainly known as saprotrophic species or causing minor damage (3, 4). The identification of *C. corticale* can be done by morphology or DNA barcoding. Morphological features are described in detail by Gregory and Waller (3). The anamorphic fruiting body can be detected after the bark of a host tree bursts open (Figures 1A,B) and releasing large numbers (30–170 million per cm^2^) of conidiospores (5). The microscopic analysis of the characteristic conidiospores (Figure 1C) in combination with the morphology of the fruiting body and the host tree species generally leads to a reliable identification (4). Another or combining method is the identification *via* DNA sequences with a focus on standard fungal barcodes, such as ribosomal internal transcribed spacer (ITS). A subsequent comparison (using BLAST algorithm) of the generated sequence with NCBI GenBank (6, 7) reveals if the specimen belongs to *C. corticale*. The distribution of *C. corticale* in North America and Europe is summarized in Table 1. In North America, the species is supposed to be indigenous. In Europe, there has been a continuous distribution since 1945 (3). The EPPO Global Database provides a distribution map with the latest changes in 2019 (https://gd.eppo.int/taxon/CRPSCO/distribution; accessed 10 Jun 2022). The typical host tree is *Acer pseudoplatanus*, but *C. corticale* is also known from other trees as hosts or dead wood, albeit much less common: *Acer campestre* L., *A. macrophyllum* Pursh, *A. negundo* L., *A. palmatum* Thunb., *A. platanoides* L., *A. rubrum* L., A. *saccharinum* L. and *A. saccharum* Marshall. Besides its typical host genus *Acer* L., *C. corticale* is also known from *Aesculus hippocastaneum* L. and *Cornus nuttallii* Audubon (8–13). It was further possible to infect some other host species by infection experiments under laboratory conditions (14).

**Table 1:** Geographical distribution of *C. corticale*.

**Continent**	**Country**	**Reference (first detection)**
North America	Canada (Ontario)	Ellis and Everhart, 1889 (2)
	United States (Colorado)	Worrall, 2022 (21)
	United States (Michigan)	Towey et al., 1932 (22)
	United States (Washington)	Worrall, 2022 (21)
	United States (Wisconsin)	Gregory and Waller, 1951 (3)
Europe	Austria	Cech, 2004 (23)
	Belgium	La News de l'OWSF, 2019 (24)
	Bulgaria	Bencheva, 2014 (12)
	Czech Republic	Koukol et al., 2015 (4)
	France	Moreau and Moreau, 1951 (25)
	Germany	Plate and Schneider, 1965 (26)
	Italy	Wilkins, 1952 (27)
	Netherlands	Verkooijen and Willems, 2016 (28)
	Norway	Spaulding, 1961 (9)
	Slovakia	Kunca et al., 2019 (29)
	Slovenia	Ogris et al., 2021 (30)
	Switzerland	Engesser et al., 2004 (31)
	United Kingdom	Gregory et al., 1949 (32)

### The sooty bark disease

**Figure 2:**
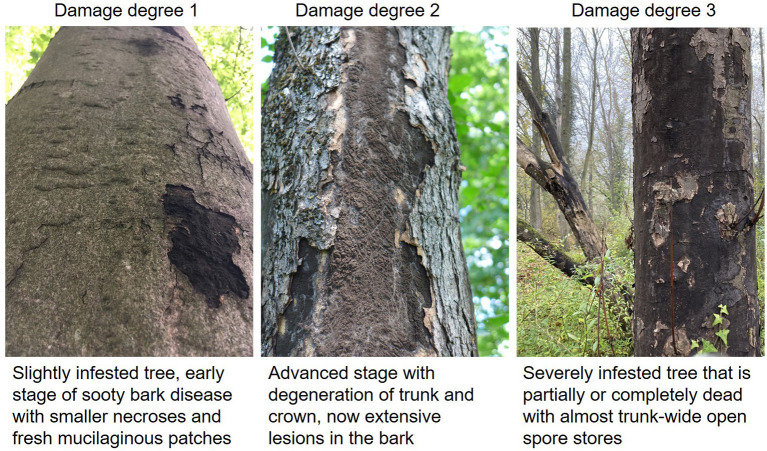
Different degrees of damage by sooty bark disease (*C. corticale*) on sycamore maple.

**Figure 3:**
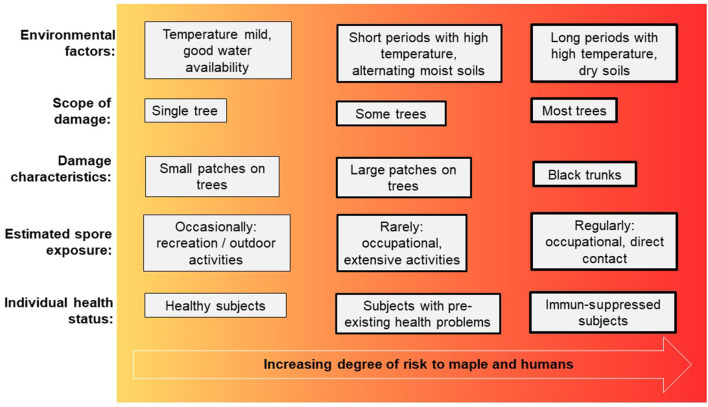
Diagram of association between factors increasing the risk for sooty bark disease in maple and respiratory health problems in humans.

The sycamore maple (*Acer pseudoplatanus*) which is known to be rejuvenating, precocious, with pioneer tree species character and good natural scattering of branches, is rather inconspicuous from the point of view to forest protection. Damage by insects to maple hardly plays a role in forest protection. For optimal growth of *Acer pseudoplatanus* a very good nutrient and water supply with high base saturation is required, while other maple species such as Norway maple (*Acer platanoides*) or field maple (*Acer campestre*) are more modest. Especially strong alternation of moisture, temporarily flooded sites and dry sites are critical for sycamore maple regarding sooty bark disease (15). Maple is one of the most valuable deciduous trees in Central Europe and large parts of Northern American, and as a typical mixed tree species, it is mostly found singly or in groups in the forests. In Germany, for example maple stands together with ash and lime tree (mixed tree species with long life span) account for about 6 % of the forest area in Germany (https://bwi.info/). Furthermore, many maple species are commonly planted in gardens as ornamental trees. The slow-growing field maple is also often cultivated as a hedge plant. In the last 20 years, due to ash dieback in Central Europe, forest areas have often been replanted with maple trees, even though the growing conditions for maples were not optimal. This makes them more susceptible to sooty bark disease, as shown by the recent waves of outbreaks since 2017, which were exacerbated by drought and extreme heat during the growing seasons (16), also see Table 1. Especially the combination of hot temperatures and lack of water is crucial for the outbreak of sooty bark disease and has been documented for Great Britain (17). Maple wood is offered as round and sawn timber as well as veneer and is mainly used in furniture construction and interior finishing and among many other uses (18). In addition, the xylem sap of sugar maple, red maple, or black maple trees is used to make maple syrup, which is widely used in Canada and the USA (19). The obvious symptoms of sooty bark disease can be divided into three degrees of damage (Figure 2). Initially, isolated dark spots occur on the trunk from which spores of the fungus break their way through the outer periderm (damage degree 1). As the damage progresses, larger parts of these fungal stromata grow together under the bark and cause a strangulation of the sap flow in the plant tissues. Together with hyphal growth in the sapwood causing white rot, the transport of assimilates and water transport is disrupted (damage degree 2). This finally leads to a decline of infested tree individuals (damage degree 3). Immediate felling of already infected trees can lead to heavy spore exposure of forest workers. Surveys (20) showed that new infection with sooty bark disease can be reduced if the infested wood is removed. It might be necessary to distinguish between debarked and barked wood, log dimensions and seasonal weather conditions in order to study the effect of removal measures and to gain more precise insights into the course of the disease. The potentially enormous quantities of spores from infected maple trees also pose a potential health risk to humans. Here, the risk increases with the frequency and intensity of spore exposure. Furthermore, the health status plays an important role, immunosuppressed people or those with pre-existing diseases are more at risk than healthy individuals. An overview of all aspects is shown in Figure 3.

### Impact on humans

Frequent and prolonged exposure to compounds triggering the immune system can cause hypersensitivity pneumonitis (HP). The disease affects the lung tissue and alveoli based on a complex type III/type IV immune response. Antigens are often presented as small particles (< 5 μm) and can be divided into five categories: animal (avian) proteins, plant proteins, drugs, low molecular weight chemicals, bacteria/molds/yeasts (microbial components) (33). Exposure to these potential antigens can occur at home or during leisure time as well as during work. HP is a rare disease with incidences about 0,9 cases per 100,000 persons (general population) per year (34); among occupationally exposed workers in United Kingdom incidence of occupational HP was shown to be 1–2 cases per million workers per year (35) and in Finland with up to 12.2 registered cases per million workers per year (36). In two current literature reviews all published cases of occupational HP by *C. corticale* were described (37, 38). Starting with the initial case report of an exposed paper mill worker (39), where subsequently all 37 workers of this paper mill were examined in a survey (40). In five workers active HP was diagnosed, nine paper miller had subclinical symptoms and in four workers serological tests (antibody precipitation, by Ochterlony test) were positive. At the same time a case report of a gardener in Berlin was described (26) and later on a case report of occupational HP in an orchid grower (41). Depending on the progress of the HP disease, three stages can be distinguished (42, 43): acute, subacute and chronic HP. During acute HP, flu-like symptoms such as chills, fever, dry cough and malaise do appear for hours to days after massive exposure to the triggering substances. After further exposure, the cough usually worsens, there is shortness of breath under physical exertion (exertional dyspnoea) and the symptoms last for days to weeks. The chronic stage usually occurs after regular, repeated exposure to often lower concentrations. Over months, the lung tissue is remodeled (so-called fibrosis), there is progressive shortness of breath, even at rest, and weight loss. In order to avoid this course of disease in the case of exposure to *C. corticale* and thus to prevent HP, a physician should be consulted if it is suspected that an occupational disease caused by *C. corticale* could arise. Because early detected HP can be treated quite successful. But diagnosis is difficult and requires various medical disciplines and examinations. Ultimately, at least six out of 10 diagnostic criteria must be fulfilled in order to diagnose HP (36). It is particularly important to establish the link between exposure and symptoms and thus means identifying the trigger. In addition, the occurrence of symptoms must be temporally related to the exposure. As an indicator of an immune response induced by a relevant exposure, the concentration of specific antibodies of the immunoglobulin G type (IgG) directed against the trigger can be determined in the patient's blood. An evaluation of the concentration of these specific antibodies can only be made in comparison to the antibody concentrations in the blood of non-exposed and exposed healthy individuals (so-called reference collectives) (44). Although *C. corticale* is known as a potential trigger of HP in exposed workers, no validated testing options are available so far. Thus, the detection of specific antibodies (IgG) against *C. corticale* is missing as an important component in the diagnosis of HP. In order to close this diagnostic gap, tools for the detection of these specific IgG antibodies in cases of suspected HP caused by *C. corticale* were developed and established at the Institute for Prevention and Occupational Medicine of the DGUV; Institute of the Ruhr University Bochum (IPA) together with the German accident insurances (UVTs) where enterprises with potentially exposed employees are insured. Reference values (so-called cut-off values) are necessary for an evaluation of the specific IgG antibody concentrations. Only in this way it can be estimated what corresponds to the “normal range” in healthy subjects and at what concentration of specific IgG antibodies represent a further indication of HP. The diagnostic tools developed by the IPA for specific antibody measurement to *C. corticale* antigens make it possible to quantitatively analyse and evaluate suspected HP cases of workers with clinical symptoms suspected of exposure to *C. corticale*. Elevated specific IgG antibody concentrations in patients' sera represent an important component in the diagnosis of HP and thus contribute to clarify the complex relationship between occupational symptoms and the suspected trigger.

## Materials and methods

### Cultivation and characterization of *C. corticale* specimens and isolates

Fifteen different strains of *C. corticale* used in this study, were collected between March 2018 and March 2020 (Table 2), and subsequently cultivated on malt extract agar (Carl Roth GmbH + Co. KG, Karlsruhe, Germany) for growth of mycelia in petri dishes. The cultivation of the strains on sterile blocks of maple wood (25 x 50 x 15 mm) on water agar in quadrangular thread bottles (1,000 ml) at 20–23°C was useful to get a sufficient amount of conidiospores within 6–10 weeks from each specimen for protein extraction. The species identification was based on fruiting body and conidiospore morphology using a confocal microscope (Axioscope, ZEISS). Genetical identification was analysed with support of ITS sequences. DNA extraction was done according to Izumitsu et al. (45). ITS1F [5'-*CTTGGTCATTTAGAGGAAGTAA*-3' (46)] and ITS4 [5'-*TCCTCCGCTTATTGATATGC*-3' (47)] were used as primers for amplifying the ribosomal DNA marker ITS (including ITS 1, 2 and 5.8S gene). The following PCR program was used: (1) initial denaturation: 94°C for 240 s, (2) denaturation: 94°C for 40 s, (3) annealing: 55°C for 40 s, (4) extension: 72°C for 50 s (number of cycles for steps 2–4: 35), (5) final extension: 72°C for 240 s. PCR products were purified with DNA Clean & Concentrator^TM^−5 (Zymo Research, Irvine, California, United States) and the DNA sequencing was implemented by LGC Genomics GmbH (Berlin, Germany). Subsequently, DNA sequences were used to identify cultivated fungal material via basic local alignment search tool for nucleotides (BLASTn, NCBI) by comparison with DNA sequences from NCBI database *via* measurements of local similarity with results of a maximal segment pair (MSP) score. When DNA sequence matches were >98%, the cultured fungal material was identified as *C. corticale*. In addition, newly identified sequences of *C. corticale* strains were uploaded to the NCBI gene bank when the quality of the DNA sequences met the five guideline quality check (48). Newly generated DNA sequences were edited with MEGA X (49) (https://www.megasoftware.net). The editing includes a trimming of both ends of the raw sequence data, a comparison of the signals in the ab1-files with the selected bases, the alignment of the forward and reverse sequences, and the construction of a consensus sequence. Subsequently, the DNA sequences were deposited in NCBI GenBank (6).

**Table 2:** Specimens and strains of *Cryptostroma corticale* used in this study.

**Specimen voucher**	**GenBank accession number**	**Origin**	**Host/Substrate**	**Collection date**	**Collector**
FVA 2176	ON786996	Germany, Baden-Wuerttemberg, Lahr	*Acer pseudoplatanus*	01 Mar 2018	Jörg Grüner
FVA 2186	OP474013	Germany, Baden-Wuerttemberg, Ludwigsburg	*Acer pseudoplatanus*	13 Apr 2018	Jörg Grüner
FVA 2190	OP474014	Germany, Baden-Wuerttemberg, Ludwigsburg	*Acer pseudoplatanus*	13 Apr 2018	Jörg Grüner
FVA 2214	ON786997	Germany, Baden-Wuerttemberg, Mannheim	*Acer pseudoplatanus*	11 Jul 2019	Jörg Grüner, Jan Tropf
FVA 2223	ON786998	Germany, Baden-Wuerttemberg, Freiburg	*Acer pseudoplatanus*	19 Mar 2020	Luisa Knauf
JKI-GFF-2019-002	ON773405	Germany, Lower Saxony, Salzgitter	*Acer pseudoplatanus*	02 Apr 2019	Rasmus Enderle, Janett Riebesehl
JKI-GFF-2019-003	OP474010	Germany, Lower Saxony, Salzgitter	*Acer pseudoplatanus*	02 Apr 2019	Rasmus Enderle, Janett Riebesehl
JKI-GFF-2019-004	ON773406	Germany, Lower Saxony, Salzgitter	*Acer pseudoplatanus*	02 Apr 2019	Rasmus Enderle, Janett Riebesehl
JKI-GFF-2019-005	OP474011	Germany, Lower Saxony, Salzgitter	*Acer pseudoplatanus*	02 Apr 2019	Rasmus Enderle, Janett Riebesehl
JKI-GFF-2019-006	ON773407	Germany, Lower Saxony, Braunschweig	*Acer pseudoplatanus*	23 May 2019	Janett Riebesehl
JKI-GFF-2019-007	OP474012	Germany, Lower Saxony, Braunschweig	*Acer pseudoplatanus*	27 Aug 2019	Rasmus Enderle, Janett Riebesehl
JKI-GFF-2019-008	ON773408	Germany, Lower Saxony, Braunschweig	*Acer pseudoplatanus*	27 Aug 2019	Rasmus Enderle, Janett Riebesehl
JKI-GFF-2019-011	ON773409	Germany, Lower Saxony, Braunschweig	*Acer pseudoplatanus*	27 Aug 2019	Rasmus Enderle, Janett Riebesehl
JKI-GFP-19-023-1	ON773410	Germany, North Rhine-Westphalia, Herford	*Acer* sp.	01 Nov 2019	Rolf Kehr
JKI-GFP-19-023-2	ON773411	Germany, North Rhine-Westphalia, Herford	*Acer* sp.	01 Nov 2019	Rolf Kehr

The specimens were identified by morphology and DNA barcoding. All sequences were uploaded in NCBI GenBank.

### Protein extraction from *C. corticale* spores

From all 15 strains of *C. corticale*, spores as well as mycelia material were prepared separately as aqueous protein extracts. Protein extraction from spores were conducted with slight modifications according to Kespohl, et al. (50): freeze-dried spore material from *C. corticale* was suspended in phosphate buffered saline (PBS) pH 7 with additional 10 mM Pefabloc (Serva, Germany) (10 mg spores per ml buffer). Suspensions were extracted by homogenization in SK38 tubes containing glass/ceramic beads in Precellys24 (PeqLab, Erlangen, Germany) for 3 cycles of 20 sec with 30 sec breaks between each cycle at 6,000 agitation per min at 4°C. The extracts were then incubated for 10 min at 4°C in an ultrasonic bath (Sonorex Super RK 255, Bandelin, Germany). Beads and cell debris were sedimented by centrifugation 5 min, at 2,820 g, 4°C. Clear supernatant was transferred into a new tube and centrifuged again at 20,000 g for 45 min at 4°C.

### Protein extraction from *C. corticale* mycelia

Protein extraction from mycelial material was prepared from fungal cultures which were grown for 10–14 days until plates were completely covered at room temperature (20–23°C) on cellulose nitrate filters covered malt extract agar plates. Mycelia material was harvested carefully and mixed with PBS, 10 mM Pefabloc in relation 1:10 (w/v). Suspensions were extracted by tissue homogenizer according to Potter-Elvehjem (VWR, Austria) for 5 min with homogenizer speed of 2,500 in ice-water according to Sander et al. (51) with modifications. The extracts were then incubated for 10 min at 4°C in an ultrasonic bath and insoluble particles were sedimented by centrifugation at 20,000 g for 45 min at 4°C. Protein concentration was measured in both, spore and mycelia extracts, using the Bradford assay (Bio-Rad, Munich, Germany) with bovine serum albumin as the standard, according to manufactures' instruction.

### SDS-Silver-PAGE

Extracts of spore and mycelia from *C. corticale* were separated according to molecular weights of the proteins by SDS-PAGE using pre-casted 12% NuPAGE gels (Invitrogen, San Diego, CA, United States) and the Xcell II mini-cell unit of Novex for 42 min at 200 V, according to the manufacturers' instructions. For calculation of molecular weights an unstained SDS PAGE protein marker from 6.5–97 kDa (Serva, Germany) was used. For each spore or mycelia extract, the same volume (30 μl) was mixed with 10 μl SDS sample buffer (Laemmli), boiled (5 min at 95°C) and applied for SDS-PAGE. In each gel pocket 25 μl of boiled protein sample was loaded, which corresponded to total protein amount of average 2.3 μg/lane (minimum 1 μg—maximum 4 μg). Silver staining of proteins was performed with small modification according to Blum et al. (52). The modifications concerned the following solutions: fixation solution (40% EtOH, 10% AcOH instead of 50% MeOH, 12%AcOH), wash solution (30% EtOH instead of 50% EtOH) and stop solution (0,5% glycine in A. bidest instead of 50% MeOH, 12%AcOH).

### Biotinylation and coupling of *C. corticale* proteins to streptavidin-ImmunoCAPs

For biotinylation, all 15 extracts of spore and mycelia material were pooled with same amounts, respectively. Protein concentration of pooled spore and pooled mycelia extract was calculated. The average molecular weight of spore and mycelia proteins were determined from SDS-Silver-PAGEs as 38 kDa for pooled spore proteins and 40 kDa for pooled mycelia proteins. Subsequently, spore and mycelia proteins were biotinylated with a 5-fold molar excess of D-biotinoyl-e-aminocaproic acid N-hydroxysuccinimide ester (NHS-Biotin) in 10 mM carbonate buffer for 2 h as described by Sander et al. (53). The final protein concentration for biotinylation was calculated for spores as 0.05 mg protein/ml and for mycelia as 0.17 mg protein/ml. Biotinylated extracts (50 μl) were loaded on Streptavidin-ImmunoCAPs (o221, Phadia, Uppsala, Sweden), as described (53) with final antigen amount of 1.9 μg spore protein and 6.1 μg mycelia protein per ImmunoCAP.

### Serum samples

In contrast to specific IgE, the reference values or cut-off values for IgG antigens are not uniform, but vary depending on the antigen. In order to be able to determine this reference value for *C. corticale* antigens, altogether 37 sera from two different groups of subjects were investigated. First, 20 sera from healthy, non-exposed subjects which were randomly selected from a non-exposed collective to determine IgG-cut off values (44). Secondly, sera from 17 healthy subjects with occupational exposure to *C. corticale* were examined.

### Ethics regulation

In order to be able to obtain sera from exposed subjects, a positive vote of the Ethics Committee of the Medical Faculty of the Ruhr University was requested and obtained (registration no.: 20–7129). With the support of the Social Insurance for Agriculture, Forestry and Horticulture (SVLFG) and other accident insurance institutions, healthy, exposed employees were recruited, and in addition to the serum, data on exposure and possible complaints caused by *C. corticale* were collected by means of questionnaires.

### Questionnaire-based exposure and respiratory symptoms induced by *C. corticale*

All participants of the exposed healthy group had reported contact to *C. corticale* in different ways. To establish *C. corticale* exposure intensity, visible infestation of maple trees both at the workplace and in private living areas were ask for. Recurrent work with wood infected with *C. corticale* was considered as regular exposure; accidental or infrequent work with wood infected with *C. corticale* was assessed as rare exposure. Potential symptoms induced by exposure to *C. corticale* were documented by asking for the following symptoms after exposure to *C. corticale*: flu-like symptoms; melalgia (joint pain), chesty cough, cough (productive with sputum), repeated fever attacks, wheezing, whistling, buzzing, chills, shortness of breath (after strenuous activities and/or at rest) and fatigue.

## Statistical analysis

Serological values were analysed and median values were given. Comparison of sIgG-values in three different exposure groups, as well as among groups with or without health complaints were analysed using GraphPad Prism 9.4.1. Calculation of significant differences among sIgG-values was done with Kruskal-Wallis-Test (multiple group comparison) for the three different exposure groups. For the two groups with or without health complaints Mann Whitney test (two group comparison) was applied.

## Results

### Quality of antigen extracts of *C. corticale* mycelia and spores

**Figure 4:**
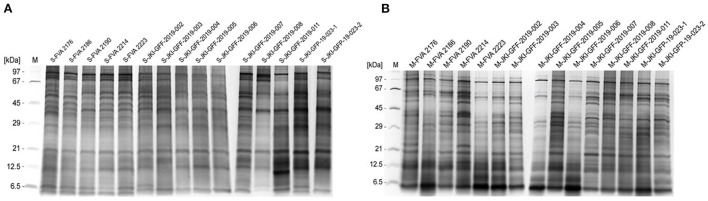
SDS-Silver-PAGE of spore extracts **(A)** and mycelia extracts **(B)** of 15 individual *C. corticale* specimens and strains which were prepared. The order of the applied extracts from left to right correspond to Table 2.

Currently no serological tests for specific IgG are available, therefore diagnostic tools for the detection of *C. corticale* spore and mycelial antigens were developed and validated. Starting with the preparation of protein extracts from genetically characterized spore and mycelium material of *C. corticale* samples (*n* = 15, Table 2). Resulting protein-antigen extracts of spore (A) and mycelium (B) were analysed by SDS-Silver-PAGE as shown in Figures 4A,B. Protein bands were detected in the molecular weight range of 5–100 kDa. In spore extracts major protein bands were detected at molecular weights of 5–6, 12, 21, 28, 35–55 and 75–100 kDa, compared to mycelia extracts with major protein bands at 5–6, 11, 18, 30–40, 57–60, and 95 kDa. The qualitative protein patterns among spores and mycelia extracts were comparable in the different strains. Accordingly, all 15 spore (Figure 4A) and mycelial (Figure 4B) extracts were pooled in same amounts, respectively. The proteins / antigens contained were subsequently biotinylated and coupled to streptavidin ImmunoCAPs.

### Characteristics of study groups

**Figure 5:**
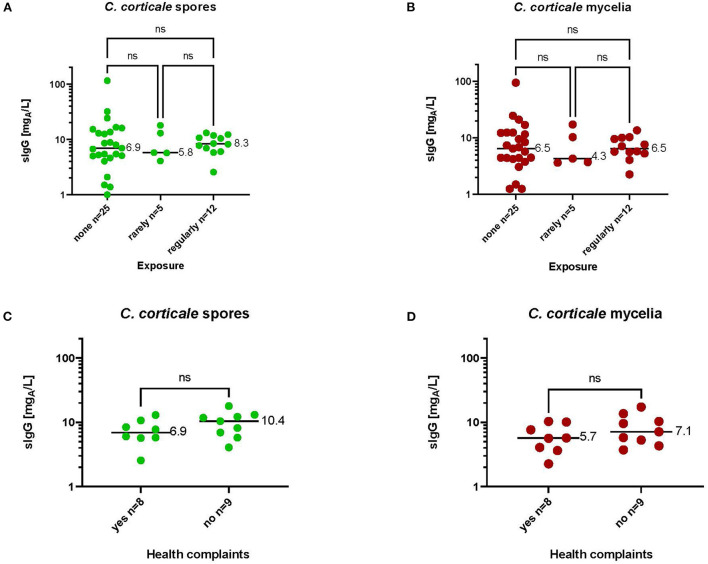
Serological sIgG-concentrations to spore **(A,C)** and mycelia **(B,D)** antigens in subjects with different exposure intensities **(A,B)** or with or without health complaints after occupational exposure **(C,D)**. Medium values of sIgG-concentrations are shown in the diagrams. Significance was calculated by Kruskal-Wallis test **(A,B)** or Mann Whitney test **(C,D)**.

In order to determine the specific IgG antibody concentration in a non-exposed group, 20 sera from a well-characterized reference group (44) were randomly selected and tested. Likewise, 17 sera from exposed but not by HP affected employees were recruited and tested as well. The characteristics of both study groups and sIgG-values to antigens from *C. corticale* are shown in Table 3. Both study groups, without and with exposure to *C. corticale*, were comparable in terms of age, gender and smoking status. Exposure as well as symptoms associated to *C. corticale* were documented by questionnaires for exposed subjects. Participants were sub-grouped according to questionnaire-based exposure frequency in non-exposed, rarely (29%) and regularly (71%) exposed. An association between health complaints and exposure to *C. corticale* has also been investigated among exposed subjects. Among rarely exposed subjects 60% mentioned health problems associated with *C. corticale* exposure versus 50% of regularly exposure. The following health complaints associated with *C. corticale* exposure were reported: flu-like symptoms (24%), chills (12%), fatigue (41%), melalgia (joint pain) (18%), dry cough (24%), wheezing, whistling, humming (6%), shortness of breath on exertion (24%). Fatigue plus at least one respiratory symptom (of dry cough, wheezing, shortness of breath) were reported in 41% of exposed healthy participants and in one participant (6%) melalgia (joint pain) has been reported as solitaire symptom. Serological IgG values to antigens from *C. corticale* spores were measured in median with 7.44 mg_A_/L in the non-exposed group compared to 8.17 mg_A_/L in exposed group. The 90% quantiles of specific IgG against spore antigens were higher in the non-exposed group (17.31 mg_A_/L) compared to the exposed group (13.04 mg_A_/L). For mycelium antigens non-exposed group has a median sIgG-value of 6.65 mg_A_/L and exposed group of 5.79 mg_A_/L. The 90% quantile for mycelia antigens was again higher in the non-exposed group (17.37 mg_A_/L) compared to the exposed group (11.65 mg_A_/L). Exposure as well as symptoms associated to *C. corticale* were considered in term of serological sIgG concentration (Figure 5). First, serological sIgG-concentration has been analysed in non-exposed, rarely and regularly exposed subject (Figures 5A,B). There was a small increase in median value of sIgG to spore antigens in those regularly exposed (8.3 mg_A_/L vs. 5.8 mg_A_/L in rarely exposed and 6.9 mg_A_/L in non-exposed). For sIgG the median value against mycelium antigens was comparable between regularly exposed and non-exposed with 6.5 mg_A_/L and a bit lower with 4.3 mg_A_/L (median value) in the sera of rarely exposed. Secondly, association of reported health complaints in exposed subjects and sIgG-values was investigated (Figures 5C,D). Here, the reported symptoms were not reflected in the serological sIgG-concentration. Rather, the opposite was observed, namely a slight increase in the median value in exposed subjects without symptoms (for spore antigens: 10.4 mg_A_/L vs. 6.9 mg_A_/L; for mycelia antigens: 5.7 mg_A_/L vs. 7.1 mg_A_/L).

**Table 3:** Characteristics of study groups.

	**Study group NON-EXPOSED (n = 20)**	**Study group EXPOSED (n = 17)**
Age: median [min-max]	44 Years [22 Y−62 Y]	47 Years [23 Y−62 Y]
Gender:	40%♀, 60%♂	35%♀, 65%♂
Smoking status:	65% Never smoker	59% Never smoker
Professions/Occupations:	Professions/Occupations without exposure to mold	Arborists, forestry workers/forestry research workers
Exposure to *C. corticale*	none	29% rarely; 71% regularly
Health complaints after *C. corticale* exposure	none	60% (rarely exposed) and 50% (regularly exposed)
*C. corticale* spores (mg_A_/L): median [min-max; 90% quantil]	7.44 [1.0–115.31; 17.31]	8.17 [2.55–17.92; 13.04]
*C. corticale* mycelia (mg_A_/L): median [min-max; 90% quantil]	6.65 [0.88–95.02; 17.37]	5.79 [2.26–17.27; 11.65]

## Discussion

We established antigen-specific IgG reference values of non-exposed healthy participants without any clinical symptoms of HP as well as of exposed healthy participants with and without suspected health complaints but without diagnosed HP from *C. corticale* spores and mycelia. The advantage of applied quantitative technique of ImmunoCAP FEIA in contrast to antibody precipitation test systems is to provide a quantitative assessment of serological IgG concentrations compared to healthy exposed and non-exposed volunteers. Quantitative test systems such as ImmunoCAP, Immulite or Sandwich ELISA-systems are usually more sensitive compared to exclusively qualitative test systems such as Ouchterlony (42). Measurement of serological IgG can therefore be a helpful instrument in the multi-faceted diagnosis of HP and allows the association between antigen exposure and immunological responses to be confirmed. Both, median sIgG values to spores (6.94 mg_A_/L and 8.17 mg_A_/L) and mycelia (6.45 mg_A_/L and 5.79 mg_A_/L) antigens of *C. corticale* were comparable in non-exposed and exposed healthy groups. Overall, the measured IgG concentrations were in the average concentration range compared to other fungal antigens measured in the cut-off study (44). There, the median sIgG value for *Rhizopus nigricans* antigens was the lowest at 1.8 mg_A_/L and the highest median value was 25.8 mg_A_/L for *Botrytis cinerea*. The comparatively high values of the 90% quantiles in the healthy unexposed group compared to the healthy exposed group is due to a very high “outliner value” in the unexposed group. This subject did not show strikingly high sIgG concentrations to any other fungal antigens, only to *C. corticale* spores and mycelium. The selection of the unexposed group was randomized and was intended to be as valid as possible, so we did not replace this 'outliner value' with another sample antigen value. Overall, the 90% quantile value in the unexposed healthy group is also in a normal range, for example, a median value of 8.3 mg_A_/L and a 90% quantile of 21.5 mg_A_/L was measured for sIgG against the mold mix Gmx6. In order to make the reference values even more representative, the number of test persons in both groups would have to be increased, which could not be realized so far.

However, what could be clearly shown in the context of the study presented here is that the sIgG concentrations for both spore and mycelial antigens do not function as exposure markers. Both groups, definitely exposed and non-exposed healthy individuals, showed comparable serological IgG concentrations to *C. corticale* antigens. The assumption that sIgG concentrations only increase when immunological response/sensitisation/disease occurs still needs to be verified by a clinical patient case. Patients with suspected HP due to *C. corticale* may have been tested for other mold antigens first due to the unavailability of *C. corticale* antigen tests, in the hope that cross-reactive structures exist in all fungi antigens. However, this assumption was already disproven in the study by Raulf et al. (44) investigating Spearman correlations between the antigen-specific IgG titers, total IgG and (unspecific) HSA-sIgG. For fungal antigens possible IgG-relevant cross-reactions (r_(Spearman)_> 0.75) were only seen for a couple of fungal species (e.g., *Penicillium chrysogenum*-*Cladosporium herbarum*-*Aspergillus fumigatus)* but not in general.

In addition to a valid diagnosis, prevention is important when working with *C. corticale*- infested material. First of all, the spread of the spores *via* the air must be avoided or minimized as far as possible. If this cannot be effectively avoided after the situational risk assessment, protective measures must be defined and implemented according to the TOP principle (T = technical; O = organizational; P = personal protective measures). This includes stopping work or increasing protective measures when symptoms of HP occurred, mechanized felling and processing and humid/damp conditions are preferable for all tree/woodwork. To minimize exposure, wood workers should rotate as often as possible and the transfer of spores through clothing into the vehicle cabins or break rooms must be avoided. For storing infested trees or wood chips, closed container/storage should be used. For personal protection FFP2/FFP3 masks with exhalation valve during motor-manual work are recommended. Work clothes should preferably be wet cleaned (washed) and shaking out should be done outdoors with respiratory protection without endangering others. A comprehensive operating instruction for the work with *C. corticale* infested woods is available by German Social Insurance for Agriculture, Forestry and Horticulture (54). Preventive strategies for working with *C. corticale* infested trees/woods are also described by other European and American institutes. The following recommendations for handling infected wood can be derived from the current knowledge in forestry practice: Firewood storage of symptom-free, barked wood from infested stands can be considered problematic, as layers of fungal stromata originate in the slowly drying stacked wood. As a result, spore contamination can also be transferred to the domestic environment. Spore production occurs exclusively within bark tissue and debarking infested logs could be a method of eliminating fungal infestation from logs. Wood without bark can be used for material or energy purposes, as it no longer poses a health risk. In daily forestry practice, however, this is hardly feasible. Therefore, it might be also possible to leave barked wood in the stand for rapid rotting processes. Another expensive and therefore rarely used method is the steaming of timber with temperatures above 70°C. Further aspects to be considered when handling infected wood are summarized in Table 4.

**Table 4:** General recommendations from operative worksite.

	**Damage degree 1**	**Damage degree 2**	**Damage degree 3**
Felling	Unproblematic (= fewer spores in the air)	Expected peak of spore production, uncertainty about hidden amount of spores under the bark (= protective measures strongly recommended)	Felling without bark after spore release (= no spore production without bark)
Processes after felling	Shredding, rotting on site in the undergrowth, combustion, removing of bark	steaming, burning on site, covered transport	high loss of timber value due to fungal degradation
Fire wood storage	Guaranteed fast drying of the split wood is important to prevent further fungal growth	not recommended	without bark possible but less value due to severe wood degradation
Protective measures for forest workers	Working during rainy periods with fewer spores in the air, wear filter mask	Felling should only be carried out under consideration of traffic safety and the risk of falling over. Mechanical felling, breathing or protective clothing, as the highest spore load is to be expected	Consider risk of breakage.

In conclusion, the sooty bark disease of maple trees is a very impressive demonstration of how tree and human health can be interrelated. Since occupational work with infected maple wood can potentially lead to a health hazard in the form of HP in wood workers, it is important to have tailored and validated diagnostic tests to detect HP as early as possible. If HP caused by sooty bark disease of infected maple trees is suspected, specific IgG tests against *C. corticale* spore and mycelium antigens were established and IgG cut-off values were determined to classify and evaluate the antigen-specific IgG results. Validation of these instruments with confirmed cases of HP should be the next step, but is certainly difficult to realize. Therefore, we offer the possibility to quantify the specific IgG concentrations in sera from suspected cases of HP due to exposure *C. corticale* with the available instruments. Request form is available at https://www.dguv.de/ipa/forschung/baproj/index.jsp.

## Data availability statement

The datasets presented in this study can be found in online repositories. The names of the repository/repositories and accession number(s) can be found in the article.

## Ethics statement

The studies involving human participants were reviewed and approved by Ethics Committee of the Medical Faculty of the Ruhr University was requested and obtained (registration no.: 20–7129). The patients/participants provided their written informed consent to participate in this study.

## Author contributions

SK and MR: conception and writing of the manuscript, evaluation and interpretation of results, initiation and coordination of the different work packages, preparation of the ethics proposal and main responsibility for scientific conception and implementation of the study. JR and JG: conception and writing of the manuscript, evaluation and interpretation of results, participation in the conception of the study and cultivation, and genetic identification of *C. corticale* material. All authors contributed to the article and approved the submitted version.

## Funding

The work was funded by the DGUV in the IPA project 145-Bioaerosols.

## Conflict of interest

The authors declare that the research was conducted in the absence of any commercial or financial relationships that could be construed as a potential conflict of interest.

## Publisher's note

All claims expressed in this article are solely those of the authors and do not necessarily represent those of their affiliated organizations, or those of the publisher, the editors and the reviewers. Any product that may be evaluated in this article, or claim that may be made by its manufacturer, is not guaranteed or endorsed by the publisher.

## References

[1] Li Q Gong X Zhang X Pi Y Long S Wu Y. Phylogeny of Graphostromatacea with two new species (*Biscogniauxia glaucae* sp. nov and Graphostroma guizhouensis sp nov) and new record of Camillea broomeana isolated in China. Arch Microbiol. (2021) 203:6119–29. 10.1007/s00203-021-02574-2 34550408

[2] Ellis JB Everhart BM. New species of hyphomycetous fungi. J Mycol. (1889) 5:68. 10.2307/3752309

[3] Gregory PH Waller S. *Cryptostroma corticale* and sooty bark disease of sycamore (*Acer pseudoplatanus*). Transact Br Mycol Soc. (1951) 34:579–IN10. 10.1016/S0007-1536(51)80043-3

[4] Koukol O Kelnarová I Cerný K. Recent observations of sooty bark disease of sycamore maple in Prague (Czech Republic) and the phylogenetic placement of *Cryptostroma corticale*. *For Path*. (2015) 45:21–7. 10.1111/efp.12129

[5] Abbey SD. The Morphology and Physiology of Cryptostroma corticale: PhD thesis. Loughborough, UK: Loughborough University of Technology. (1978)

[6] Benson DA Cavanaugh M Clark K Karsch-Mizrachi I Ostell J Pruitt KD. GenBank. Nucleic Acids Res. (2018) 46:41–7. 10.1093/nar/gkx1094 29140468 PMC5753231

[7] Raja AH Miller AN Pearce CJ Oberlies NH. Fungal identification using molecular tools: a primer for the natural products research community. J Nat Prod. (2017) 80:756–70. 10.1021/acs.jnatprod.6b01085 28199101 PMC5368684

[8] Moreau C Moreau M. Nouvelles observationes sur le deperissement des erables. Bulletin de la Société mycologique de France. (1954) 7:66–7.

[9] Spaulding P. Foreign Diseases of Forest Trees of the World: An Annotated List. Agriculture Handbook No 197, Washington DC: US Government Printing Office. (1961).

[10] Ohman JH Kessler KJ Meyer GC. Control of *Cryptostroma corticale* on stored sugar maple pulpwood. Phytopathology. (1969) 59:871.

[11] Young CW. Sooty bark disease of sycamore. Arboricult Leaflet. (1978) 3:1–8.33478566

[12] Bencheva S. First report of *Cryptostroma corticale* (Ellis & Everh.) PH Greg. &* S Waller on Acer platanoides L in Bulgaria. Silva Balc*. (2014) 15:101–4.

[13] Forest Pathology,. (2022) *Sooty Bark Disease*. Available online at: https://forestpathology.org/canker/sooty-bark-maple/ (accessed June 19, 2022).

[14] Dickenson S. Biology of Cryptostroma corticale and the Sooty Bark Disease of Sycamore. [PhD thesis]. London, UK: University of London. (1980)

[15] Roloff A Weisgerber H Lang UM Stimm B. Bäume Mitteleuropas. Von Aspe bis Zirbelkiefer. Mit den Porträts aller Bäume des Jahres von 1989 bis 2010. Weinheim, Germany: Wiley-VCH (2010). p. 3–28.

[16] Grüner J Berens A Delb H. Die Ahorn-Rußrindenkrankheit in Südwestdeutschland: Gefahren, Prognose und Empfehlungen. In: Waldschutzinfo. (2020) 2:1–8.

[17] Gibbs JN. Fifty years of sooty bark disease of sycamore. Quart J Forest. (1997) 91:215–21.

[18] Höllerl S. Mosandl R. Der Bergahorn im Bergmischwald-unübertroffen in seinem Verjüngungspotenzial, Beiträge zum Bergahorn, LWF Wissen. (2009) 62:24–30.

[19] Randall JA. Maple Syrup Production. In: ISU Forestry Extension. p. 1–4.

[20] Stange L Theisinger O Lieffertz A Theisinger W Niesar M. Ergebnisse aus der Abfrage zum Vorkommen der Rußrindenerkrankung an Ahorn in NRW 2021. In: Waldschutzinfo. (2022) 7:1–4.

[21] Worrall JJ. Sooty-Bark Disease of Maple (2022). Available online at: https://forestpathology.org/canker/sooty-bark-maple/

[22] Towey JW. Severe bronchial asthma apparently due to fungus spores found in maple bark. JAMA. (1932) 99:453. 10.1001/jama.1932.0274058002100525996397

[23] Cech T. Bemerkenswerte Krankheiten in 2004. Forstschutz Aktuell. (2004) 32:31–4.

[24] La News de l'OWSF (Avril 2019). Présence de la suie de l'érable sur le territoire wallon [cited 2019] (2019). Available from: http://owsf.environnement.wallonie.be/fr/26-04-2019-owsf-news-avril-2019.html?IDC=5792&IDD=6073

[25] Moreau C Moreau M. La ‘Suie' des Sycamores a Paris. Bulletin de la Société mycologique de France. (1951) 67:404–18.

[26] Plate HP Schneider R. Ein Fall von asthmaartiger Allergie, verursacht durch den Pilz *Cryptostroma corticale*. *Nachr Dtsch Pflanzenschutzd*. (1965) 17:100–1.

[27] Wilkins VE. Report of the technical working party: edited by european plant protection organisation (EPPO). European Plant Protection Organisation (EPPO). Paris (1952).

[28] Verkooijen R Willems J. Roetschorsziekte nu ook in Nederland. TBV - Tijdschr Bedrijfs- en Verzekeringsgeneeskd. (2016) 24:166–7. 10.1007/s12498-016-0065-1

[29] Kunca A Zúbrik M Nikolov C Leontovyè R Galko J Vakula Je. The Occurrence of the Pathogenic Fungi Cryptostroma corticale, Prosthecium pyriforme and Eutypella parasitica on Acer pseudoplatanus from 2017 to 2019 in Slovakía: Editura Universitã“tefan cel Mare” (Ed.): Recent Changes in Forest Insects and Pathogens Significance: IUFRO 7. 03.10. Suceava: Methodology of forest insect and disease survey in Central Europe Meeting 2019 (2019).

[30] Ogris N Brglez A Piškur B. Drought stress can induce the pathogenicity of cryptostroma corticale, the causal agent of sooty bark disease of sycamore maple. Forests. (2021) 12:377. 10.3390/f12030377

[31] Engesser R Forster B Meier F Odermatt O. Forstschutzsituation 2003 in der Schweiz. AFZ-Der Wald. (2004) 59:385–7.

[32] Gregory PH Peace TR Waller S. Death of sycamore trees associated with an unidentified fungus. Nature. (1949) 164:275. 10.1038/164275a018139366

[33] Sennekamp H-J. Extrinsic Allergic Alveolitis: Hypersensitivity Pneumonitis. Munich: Dustri-Verl. Feistle (2004). p. 510.

[34] Solaymani-Dodaran M West J Smith C Hubbard R. Extrinsic allergic alveolitis: incidence and mortality in the general population. QJM. (2007) 100:233–7. 10.1093/qjmed/hcm008 17307752

[35] Barber CM Wiggans RE Carder M Agius R. Epidemiology of occupational hypersensitivity pneumonitis; reports from the SWORD scheme in the UK from 1996 to 2015. Occup Environ Med. (2017) 74:528–30. 10.1136/oemed-2016-103838 27919062 PMC5520266

[36] Quirce S Vandenplas O Campo P Cruz MJ Blay F de Koschel D. Occupational hypersensitivity pneumonitis: an EAACI position paper. Allergy. (2016) 71:765–79. 10.1111/all.12866 26913451

[37] Braun M Klingelhöfer D Groneberg DA. Sooty bark disease of maples: the risk for hypersensitivity pneumonitis by fungal spores not only for woodman. J Occup Med Toxicol. (2021) 16:2. 10.1186/s12995-021-00292-5 33478566 PMC7819180

[38] Madsen AM Crook B. Occupational exposure to fungi on recyclable paper pots and growing media and associated health effects - A review of the literature. Sci Total Environ. (2021) 788:147832. 10.1016/j.scitotenv.2021.147832 34034170

[39] Emanuel DA Lawton BR Wenzel FJ. Maple-bark disease. Pneumonitis due to Conidiosporium corticale. N Engl J Med. (1962) 26:333–7. 10.1056/NEJM196202152660704 13890068

[40] Wenzel FJ Emanuel DA. The epidemiology of maple bark disease. Arch Environ Health. (1967) 14:385–9. 10.1080/00039896.1967.10664759 4951671

[41] Shepherd GM Michelis MA Macris NT Smith JP. Hypersensitivity pneumonitis in an orchid grower associated with sensitivity to the fungus *Cryptostroma corticale*. *Ann Allergy*. (1989) 62:522–5. 2735559

[42] Nogueira R Melo N. Novais e Bastos H Martins N Delgado L Morais A. Hypersensitivity pneumonitis: antigen diversity and disease implications. Pulmonology. (2019) 25:97–108. 10.1016/j.pulmoe.2018.07.003 30126802

[43] Costabel U Miyazaki Y Pardo A Koschel D Bonella F Spagnolo P. Hypersensitivity pneumonitis. Nat Rev Dis Primers. (2020) 6:65. 10.1038/s41572-020-0191-z 32764620

[44] Raulf M Joest M Sander I Hoffmeyer F Nowak D Ochmann U. Update of reference values for IgG antibodies against typical antigens of hypersensitivity pneumonitis. Allergo J. (2019) 28:192–203. 10.1007/s40629-019-0099-x

[45] Izumitsu K Hatoh K Sumita T Kitade Y Morita A Tanaka C. Rapid and simple preparation of mushroom DNA directly from colonies and fruiting bodies for PCR. Mycoscience. (2012) 53:396–401. 10.1007/S10267-012-0182-3

[46] Gardes M Bruns TD ITS. primers with enhanced specificity for basidiomycetes–application to the identification of mycorrhizae and rusts. Mol Ecol. (1993) 2:113–8. 10.1111/j.1365-294X.1993.tb00005.x 8180733

[47] White TJ Bruns T Lee S Taylor J. Amplification and Direct Sequencing of Fungal Ribosomal RNA Genes for Phylogenetics. In:Innis MA Gelfand DH Sninsky JJ White TJ, editor, *PCR Protocols: A Guide to Methods and Applications*, Academic Press, New York. p. 315–322.

[48] Nilsson RH Tedersoo L Abarenkov K Ryberg M Kristiansson E Hartmann M. Five simple guidelines for establishing basic authenticity and reliability of newly generated fungal ITS sequences. MycoKeys. (2012) 4:37–63. 10.3897/mycokeys.4.3606

[49] Kumar S Stecher G Li M Knyaz C Tamura K MEGA X. Molecular evolutionary genetics analysis across computing platforms. Mol Biol Evol. (2018) 35:1547–9. 10.1093/molbev/msy096 29722887 PMC5967553

[50] Kespohl S Maryska S Zahradnik E Sander I Brüning T Raulf-Heimsoth M. Biochemical and immunological analysis of mould skin prick test solution: current status of standardization. Clin Exp Allergy. (2013) 43:1286–96. 10.1111/cea.12186 24152161

[51] Sander I Zahradnik E van Kampen V Kespohl S Stubel H Fischer G. Development and application of mold antigen-specific enzyme-linked immunosorbent assays (ELISA) to quantify airborne antigen exposure. J Toxicol Environ Health A. (2012) 75:1185–93. 10.1080/15287394.2012.707603 22994572

[52] Blum H Beier H Gross HJ. Improved silver staining of plant-proteins, RNA and DNA in Polyacrylamid gels. Electrophoresis. (1987) 8:93–9. 10.1002/elps.1150080203

[53] Sander I Kespohl S Merget R Goldscheid N Degens P Brüning T. A new method to bind allergens for the measurement of specific IgE antibodies. Int Arch Allergy Immunol. (2005) 136:39–44. 10.1159/000082583 15591812

[54] SVLFG Sozialversicherung für, Landwirtschaft, Forsten und, Gartenbau. B.01.18 Schutzmaßnahmen bei Tätigkeiten an Ahorn mit Rußrindenkrankheit. (2019). Available online at: https://cdn.svlfg.de/fiona8-blobs/public/svlfgonpremiseproduction/bbb58ebb5c9760d3/3aa2ce9f2c9a/b_01_18-bio-arbeitsstoffe-russrindenkrankheit. (accessed: June 15, 2019)

